# Supplementary material for: Vaginal hormone-free moisturising cream is not inferior to an estriol cream for treating symptoms of vulvovaginal atrophy: Prospective, randomised study
Source: PLoS One. 2022 May 12;17(5):e0266633. doi: 10.1371/journal.pone.0266633 (PMC9098008; doi:10.1371/journal.pone.0266633)
Supplement: S1 File — (PDF) [file pone.0266633.s001.pdf]

1 TITLE PAGE

Clinical Trial Protocol (in accordance with ICH E6)

Version: Final 1.0

Prospective, open-label, multicenter, multinational, randomized trial to investigate the non-inferiority of treatment with Vagisan® Moisturising Cream in comparison to an Estriol containing cream in a panel of post-menopausal women suffering from the symptoms of "vulvovaginal dryness" in a parallel group design

**Short Title**	Investigation of non-inferiority of treatment of "vulvovaginal dryness" with Vagisan® Moisturising Cream in comparison to an Estriol containing cream	
**Investigational Product**	Vagisan® FeuchtCreme (Vagisan® Moisturising Cream) (medical device)	
**Intended Indication**	"Vulvovaginal dryness"	
**Trial Design**	Prospective, open-label, multicenter, multinational, randomized trial with parallel group design	
**Phase of Development**	Phase IV	
**Planned Trial Period**	First Patient In:<br>NOV 2016	Last Patient Out:<br>JUN 2017
**Sponsor**	Dr. August Wolff GmbH & Co. KG Arzneimittel<br>Sudbrackstraße 56, 33611 Bielefeld, Germany	
**Sponsor Code**	VFCr-12/2015	
**EudraCT-No.**	2016-002199-28	
**CRO**		
**Coordinating Investigator for Switzerland / overall**		
**Coordinating Investigator for Germany**		

2 SYNOPSIS

Title of the Trial	Prospective, open-label, multicenter, multinational, randomized trial to investigate the non-inferiority of treatment with Vagisan® Moisturising Cream in comparison to an Estriol containing cream in a panel of post-menopausal women suffering from the symptoms of "vulvovaginal dryness" in a parallel group design
Short Title	Investigation of non-inferiority of treatment of "vulvovaginal dryness" with Vagisan® Moisturising Cream in comparison to an Estriol containing cream
Clinical Phase	Phase IV
EudraCT Number	2016-002199-28
Coordinating Investigator Switzerland / overall	
Coordinating Investigator Germany	
Trial Center(s)	
Main References	**Bachmann G,** Urogenital ageing: an old problem newly recognized. Maturitas 22 Suppl. S1-S5, 1995<br>**Biglia N et al,** Low-dose vaginal estrogens or vaginal moisturizer in breast cancer survivors with urogenital atrophy: a preliminary study. Gynecological Endocrinology 26(6):404-12, 2010<br>**Stute P et al,** Efficacy and safety of non-hormonal remedies for vaginal dryness: open, prospective, randomized trial. Climacteric. 18(4):582-9, 2015<br>**NAMS (North American Menopause Society),** Management of symptomatic vulvovaginal atrophy: 2013 position statement of the North American Menopause Society. Menopause 20(9):888-902, 2013
Trial Period (planned)	First Patient in: NOV 2016<br>Last Patient out: JUN 2017

Objectives	The aim of the study is to gain further experience with regard to the performance of the medical device Vagisan® Moisturising Cream in comparison to an Estriol containing cream.<br>**<u>Primary Objective</u>**<br>The main objective of this trial is to investigate the non-inferiority of the treatment of "vulvovaginal dryness" with Vagisan® Moisturising Cream in comparison to an Estriol containing cream.<br>The comparison will be based on the subjective assessment of the symptoms of "vulvovaginal dryness" (**Total Severity Score**: sum score of the single subjective symptom parameters dryness, itching, burning and pain unrelated to sexual intercourse).
	**<u>Secondary Objectives</u>**<br><u>Efficacy Assessments:</u><br>• Severity scoring for each of the subjective symptoms (dryness, itching, burning and pain unrelated to sexual intercourse)<br>• Impairment of daily life due to each subjective symptom<br>• Mean sum score for severity of the subjective symptoms and impairment of daily life due to each subjective symptom<br>• Severity scoring for dyspareunia (if sexually active)<br>• Impairment of daily life due to dyspareunia (if sexually active)<br>• Overall impairment of daily life due to the condition "vaginal dryness", including subgroup analysis of patients with mild, moderate or severe impairment<br>• Vaginal Health Index (objective assessment of vaginal findings)<br>• Comparison of Vagisan® Moisturising Cream to Estriol containing cream on Area under the Curve (AUC) over the course of the study for all above given parameters.<br>• Vaginal status of *Lactobacillus* flora<br>• Global judgement of efficacy by the Investigator<br>• Global judgement of efficacy by the patient<br><u>Safety Assessments:</u><br>• Global judgement of tolerability by the Investigator<br>• Global judgement of tolerability by the patient<br>• Safety parameters (adverse events, concomitant medication)<br><u>Further Assessments:</u><br>• Pre-treatment of "vulvovaginal dryness" and satisfaction with the respective product<br>• Urinary incontinence<br>• Questionnaire about satisfaction with the respective test product<br>• Dose and frequency of the treatment with the respective test product
Trial Design	Prospective, open-label, multicenter, multinational, randomized trial with parallel group design
Number of Patients (planned)	172 female patients will be randomized in this trial. Taking into account an anticipated drop-out rate of 25%, 138 patients are expected to complete the trial (69 in each of the two arms of the trial).

Diagnosis and Main Criteria for Inclusion	Post-menopausal women with symptoms of "vulvovaginal dryness"
Investigational Product	Vagisan® FeuchtCreme (Vagisan® Moisturising Cream) (medical device)
Reference Product	Ovestin® 1 mg Creme (Germany)/ Ovestin® Crème (Switzerland) (medicinal product) – in the following: Ovestin®
Method and Mode of Application:	The investigational product and the reference product will be applied according to the respective package leaflet; to be precise the products will be applied as follows:<br>2.5 g of the investigational product Vagisan® Moisturising Cream will be applied intravaginally, once daily in the evening. After improvement of the symptoms the frequency may be reduced by the patient as needed. In addition 0.5 g (1 fingertip unit) may be applied to the outer genital area as needed (also several times per day).<br>0.5 g of the reference product Ovestin® will be applied intravaginally once daily in the evening for the first 3 weeks. Subsequently, the frequency will be reduced to twice a week for the last 3 weeks for all patients in this treatment group.<br>All applications will be performed by the patients themselves at home.
Trial Course	On Visit 1 objective and subjective assessments will be performed in the trial center and a vaginal smear will be taken to investigate the *Lactobacillus* flora status. Afterwards patients will be instructed by the Investigator or the Study Nurse how to apply the test products and they will receive the first part of the patient diary.<br>After 3 weeks (Visit 2) the patients will return to the trial centers for the assessment of objective and subjective parameters. They will receive the second part of the patient diary. On the final visit at the end of 6 weeks (Visit 3), objective and subjective assessments will be performed in the trial center, a vaginal smear will be taken for re-analysis and patients will be asked to complete a questionnaire to assess their satisfaction with the respective treatment.<br>On all visits the patients will be asked about concomitant therapies; on Visit 2 and 3 they will be asked about adverse events.
Duration:	Duration of Treatment: 6 weeks per patient<br>Duration of Trial: 8 months
Efficacy Assessment(s):	The severity of each of the subjective symptoms **dryness, itching, burning and pain unrelated to sexual intercourse** will be assessed by the patient on Visits 1, 2 and 3 and once weekly at home in a patient diary according to the following scale (for each parameter separately):<br>0 = none<br>1 = mild<br>2 = moderate<br>3 = severe<br>4 = very severe

	In addition, the **impairment of each subjective symptom** (dryness, itching, burning and pain unrelated to sexual intercourse) on daily life will be assessed on Visits 1, 2 and 3 and once weekly in a patient diary by using a Visual Analogue Scale (VAS):<br>0 = no impairment<br>10 = very pronounced impairment<br>The **dyspareunia** (if sexually active) will be assessed by the patient on Visits 1, 2 and 3 according to the following scale:<br>0 = none<br>1 = mild<br>2 = moderate<br>3 = severe<br>4 = very severe<br>The **impairment** of daily life due to dyspareunia (if sexually active) will be assessed on Visits 1, 2 and 3 by using a VAS scale:<br>0 = no impairment<br>10 = very pronounced impairment<br>**Overall impairment of daily life due to the condition "vaginal dryness"** will be assessed on Visits 1, 2 and 3 by using a Visual Analogue Scale (VAS)<br>0 = no impairment<br>10 = very pronounced impairment<br>For the subgroup analysis the **overall impairment** will be categorized according to the scale:<br>0.1 to 3.3 = mild<br>3.4 to 6.6 = moderate<br>6.7 to 10 = severe<br>In case of too few patients per subgroup, categories may be combined.

The objective status of the vagina will be assessed by the Investigator on Visits 1, 2 and 3 according to the **Vaginal Health Index** (Bachmann, 1995). Each of the five criteria - elasticity, fluid secretion, pH, epithelial mucosa and moisture - will be graded from 1 (worst) to 5 (best) and then be summed up, so that the minimum score will be 5 (worst) and the maximum score will be 25 (best):

	1	2	3	4	5
Overall Elasticity	None	Poor	Fair	Good	Excellent
Fluid secretion type and consistency	None	Scant, thin yellow	Superficial, thin white	Moderate, thin white	Normal (white flocculent)
pH	≥ 6.1	5.6–6.0	5.1–5.5	4.7–5.0	≤ 4.6
Epithelial mucosa	Petechiae noted before contact	Bleeds with light contact	Bleeds with scraping	Not friable, thin mucosa	Not friable, normal mucosa
Moisture	None, mucosa inflamed	None, mucosa not inflamed	Minimal	Moderate	Normal

A vaginal smear will be taken on Visits 1 and 3 to assess the status of ***Lactobacillus*** flora (normal range, increased, decreased).

The **global judgement of efficacy** will be assessed by the Investigator and the patient on Visit 3 according to the following scale:

1 = very good

2 = good

3 = moderate

4 = poor

Safety Assessment	The **global judgement of tolerability** will be assessed by the Investigator and the patient on Visit 3 according to the following scale:<br>1 = very good<br>2 = good<br>3 = moderate<br>4 = poor<br>**Adverse events** will be documented on Visits 2 and 3 (and in the patient diary, if applicable)<br>**Concomitant medication** will be documented on Visits 1, 2 and 3 (and in the patient diary, if applicable)
Further Assessments	The **satisfaction** with pre-treatment for "vaginal dryness" will be assessed by the patient on Visit 1 by using the following scale:<br>1 = very good<br>2 = good<br>3 = moderate<br>4 = poor<br>The **urinary incontinence** will be assessed by the patient at each visit:<br>Visit 1: yes/no question<br>Visit 2 and Visit 3: improvement/worsening/no change<br>**Satisfaction with the respective treatment** will be assessed by the patient on Visit 3 by a questionnaire.<br>**Dose and frequency of the treatment** with the respective test product will be assessed daily in the patient diary.
Plan for Data Analysis / Statistics:	Primary endpoint:<br>The **Total Severity Score** is defined as the sum of the single parameters dryness, itching, burning and pain unrelated to sexual intercourse, each scored from 0 = none to 4 = very severe. In total a range of 0 = no complaints to 16 = very severe complaints is possible.<br>Differences to Baseline (Visit 1) of the Total Severity Score assessed after six weeks of treatment (Visit 3) serve as the primary endpoint for the test of non-inferiority.<br><br>Secondary endpoint(s):<br><u>Efficacy Assessments:</u><br>• Severity scoring for each of the subjective symptoms (dryness, itching, burning and pain unrelated to sexual intercourse) at each visit and once weekly<br>• AUC for severity scoring for each of the subjective symptoms over all assessment time points (at each visit and once weekly)<br>• Impairment of daily life due to each subjective symptom at each visit and once weekly<br>• AUC for impairment of daily life due to each subjective symptom over all assessment time points (at each visit and once weekly)

	• Mean sum score for severity of the subjective symptoms and impairment of daily life due to each subjective symptom at each visit and once weekly. For better interpretation severity scoring will be recalculated to a scale from 0 to 10 resulting in: mean sum score = (severity scoring + impairment of daily life) / 8<br>• AUC for this mean sum score over all assessment time points (at each visit and once weekly)<br>• Severity scoring for dyspareunia (if sexually active) at each visit<br>• AUC for severity scoring for dyspareunia (if sexually active) over all assessment time points (at each visit)<br>• Impairment of daily life due to dyspareunia (if sexually active) at each visit<br>• AUC for impairment of daily life due to dyspareunia (if sexually active) over all assessment time points (at each visit)<br>• Overall impairment of daily life due to the condition “vaginal dryness”, including a subgroup analysis of patients with mild, moderate and severe overall impairment at each visit<br>• AUC for overall impairment of daily life over all assessment time points (at each visit)<br>• Vaginal Health Index, for each parameter, and as sum score over objective assessment of vaginal findings at each visit<br>• AUC for Vaginal Health Index (for each parameter, and as sum score) over all assessment time points (at each visit)<br>• Vaginal status of Lactobacillus flora on Visit 1 and on Visit 3<br>• Global judgment of the efficacy by the Investigator on Visit 3<br>• Global judgment of the efficacy by the patient on Visit 3<br>Safety Assessments:<br>• Global judgment of the tolerability by the Investigator on Visit 3<br>• Global judgment of the tolerability by the patient on Visit 3<br>• Safety parameters (adverse events, concomitant medication)<br>Further Assessments:<br>• Pre-treatment of "vulvovaginal dryness" and satisfaction with the respective product on Visit 1<br>• Urinary incontinence at each visit<br>• Questionnaire about satisfaction with the respective test product on Visit 3<br>• Dose and frequency of the treatment with the respective test product
Statistical Methods	For primary objective:<br>• Independent t-test (one-sided) to demonstrate non-inferiority of the primary parameter<br>• Once non-inferiority is proven, the data will be analyzed with independent t-test (one-sided) for superiority using hierarchical testing methods<br>• If the assumptions of the t-test do not hold (normality rejected by Shapiro-Wilk Test) Mann-Whitney Wilcoxon test will be used instead<br>• As analysis to account for the center effect a mixed model with the fixed factor treatment and the random factors center and treatment-by-center will be done<br>• ANCOVA with Baseline as covariate and (depending on the results of the mixed model) including center effect

	• Descriptive statistics<br>For secondary objectives:<br>• Independent t-test (two sided) for AUC<br>• Descriptive statistics for all objectives

2.1 Flow Chart

	Visit 1	Treatment	Visit 2	Treatment	Visit 3
	Day 1	Day 1-21	Day 22 (+/-2 days)	Day 22-42	Day 43 (+/- 4 days)
Informed consent	X				
Demography and medical history	X				
Inclusion and exclusion criteria	X				
Subjective assessment of dryness, itching, burning, pain unrelated to sexual intercourse (Scoring)	X	X Once weekly	X	X Once weekly	X
Assessment of overall impairment of daily life due to the condition “vaginal dryness” (VAS)	X		X		X
Gynecological examination (in view of exclusion criteria)	X				
Blood pressure and pulse rate	X		X		X
Body Mass Index	X				
Vaginal Health Index	X		X		X
Vaginal Smear for *Lactobacillus* status	X				X
Documentation of pre-treatment of "vulvovaginal dryness" and satisfaction with the respective product	X				
Assessment of impairment of daily life due to each subjective symptom (VAS)	X	X Once weekly	X	X Once weekly	X
Assessment of dyspareunia (if sexually active) (Scoring) and impairment on daily life (VAS)	X		X		X
Assessment of urinary incontinence	X		X		X
Assessment of global judgement of efficacy (patient and Investigator)					X
Assessment of global judgement of tolerability (patient and Investigator)					X
Dose and frequency of the treatment with the respective study product		X daily		X daily	
Questionnaire for satisfaction with the respective treatment					X
Discussion of further treatment					X
Dispensing (D) and return (R) of patient's diary	D		R/D		R

	**Visit 1**	**Treatment**	**Visit 2**	**Treatment**	**Visit 3**
	Day 1	Day 1-21	Day 22 (+/-2 days)	Day 22-42	Day 43 (+/- 4 days)
Dispensing (D) and return (R) of test product	D				R
Documentation of adverse events		X (If applicable, diary)	X	X (If applicable, diary)	X
Concomitant therapies	X				
Change of concomitant therapies		X (If applicable, diary)	X	X (If applicable, diary)	X

Table 2.1 Flow Chart
